# The role of c-Jun for beating cardiomycyte formation in prepared embryonic body

**DOI:** 10.1186/s13287-023-03544-9

**Published:** 2023-12-18

**Authors:** Lide Su, Guofu Zhang, Lili Jiang, Chao Chi, Bing Bai, Kai Kang

**Affiliations:** 1 Department of Cardiovascular Surgery, The First Affiliated Hospital of Harbin Medical University, Harbin, 150001 Heilongjiang China; 2 Department of Pediatric Dentistry, The First Affiliated Hospital of Harbin Medical University, Harbin, 150001 Heilongjiang China; 3 Department of Cardiology, The First Affiliated Hospital of Harbin Medical University, Harbin, 150001 Heilongjiang China

**Keywords:** C-Jun, Cardiac differentiation, Mesoderm, Endoderm, Embryonic body

## Abstract

**Background:**

Morbidity and mortality associated with cardiovascular diseases, such as myocardial infarction, stem from the inability of terminally differentiated cardiomyocytes to regenerate, and thus repair the damaged myocardial tissue structure. The molecular biological mechanisms behind the lack of regenerative capacity for those cardiomyocytes remains to be fully elucidated. Recent studies have shown that c-Jun serves as a cell cycle regulator for somatic cell fates, playing a key role in multiple molecular pathways, including the inhibition of cellular reprogramming, promoting angiogenesis, and aggravation of cardiac hypertrophy, but its role in cardiac development is largely unknown. This study aims to delineate the role of c-Jun in promoting early-stage cardiac differentiation.

**Methods:**

The c-Jun gene in mouse embryonic stem cells (mESCs) was knocked out with CRISPR-Cas9, and the hanging drop method used to prepare the resulting embryoid bodies. Cardiac differentiation was evaluated up to 9 days after c-Jun knockout (ko) via immunofluorescence, flow cytometric, and qPCR analyses.

**Results:**

Compared to the wild-type control group, obvious beating was observed among the c-Jun-ko mESCs after 6 days, which was also associated with significant increases in myocardial marker expression. Additionally, markers associated with mesoderm and endoderm cell layer development, essential for further differentiation of ESCs into cardiomyocytes, were also up-regulated in the c-Jun*-*ko cell group.

**Conclusions:**

Knocking out c-Jun directs ESCs toward a meso-endodermal cell lineage fate, in turn leading to generation of beating myocardial cells. Thus, c-Jun plays an important role in regulating early cardiac cell development.

**Supplementary Information:**

The online version contains supplementary material available at 10.1186/s13287-023-03544-9.

## Introduction

Myocardial infarction (MI) is a leading cardiovascular disease, with high severity, mortality, and poor prognosis. Adult cardiomyocytes are terminally differentiated, meaning they are incapable of regeneration or self-repair once damaged. As a result, even after extensive interventional therapy, treating severe heart failure may require heart transplantation, which is limited by the shortage of heart donors [1–4]. Thus, numerous studies have been performed over the years to find an effective treatment, involving replacement of damaged cardiomyocytes, of which embryonic stem cells (ESCs), owing to their totipotency, have been the most popular cell candidate [5, 6]. ESC-derived cardiomyocytes have been found to share structural and functional similarities with adult cardiomyocytes, making them a promising therapy for cardiac regeneration [7]. Recent studies have shown these cardiomyocytes are able to successfully replace damaged and necrotic cell when transplanted into infarcted hearts. Additionally, they could be used to build an in vitro heart disease model to aid in further clinical research [8, 9]. Although several cardiac differentiation schemes have been developed with increased efficiency, involving the use of a variety of small molecular compounds to induce ESC differentiation into cardiomyocytes, the overall differentiation efficiency remains suboptimal, and the quality of the resulting cardiomyocytes are not consistent [10–12]. Therefore, multiple challenges are present for applying ESC-derived cardiac cells into clinical practice, particularly with determining the key regulatory factors responsible for promoting the differentiation from ESCs into mesodermal cells, which then serves as the basis for subsequent cardiomyocyte development, as well as ensuring that the resulting homogeneous mature cardiomyocytes are obtained [13–15].

AP-1 is a transcription factor critical for cell growth and differentiation, consisting of either a highly conserved homo- or heterodimer of Jun, FOS, JDP, and ATF proteins [16, 17]. C-Jun, a vital component of AP-1, has been implicated in embryonic development, in which it is involved in maintaining somatic cell states, inhibiting their reprogramming into pluripotent cells [18–21]. Increasing evidence have also demonstrated links between c-Jun and the occurrence of multiple heart diseases [22, 23]. Studies have revealed that c-Jun upregulation in rats leads to cardiac myoblast proliferation [24], and rats without this gene develop cardiac outflow tract malformations similar to those found in human arterial stem disease [25]. Disease models have also shown that c-Jun activation is a necessary regulatory factor for cardiac hypertrophy [22, 23]. However, other studies have shown c-Jun protecting the heart from pathological remodeling under adverse stress [26]. All these findings therefore suggest that c-Jun plays an important role in cardiomyocyte development and pathophysiological processes, though the specific molecular mechanisms involved are still largely unknown.

This study aims to further clarify the role that c-Jun plays in cardiomyocyte differentiation. We knocked out the gene within mouse embryonic stem cells (mESCs) through CRISPR-Cas9 technology [27]. Embryoid bodies (EBs) were formed and cultured within differentiation medium under suspension for 6 days. These EBs were then subjected to adherent cell culturing, but with continued differentiation, from Days 7–9. We found large numbers of beating cells in c-Jun-knockout (ko) EBs on Day 6, which were confirmed to be cardiomyocytes under RT-qPCR. By contrast, wild-type (wt) EBs, even on Day 9, only contained weak beating cells. Thus, c-Jun serves as a negative regulator in the early stages of heart development, in which its silencing results in the promotion of ESC differentiation into mesodermal and endodermal cells, and subsequently cardiomyocytes.

## Materials and methods

### Cell culture

The mESC line, derived from C57 mice, was provided by Duanqing Pei’s laboratory (Guangzhou Institute of Biomedicine and Health, Chinese Academy of Sciences). ESCs were cultured, feeder-free, in N2B27-2i medium, consisting of 50% (v/v) DMEM (Hyclone), 50% (v/v) knock out DMEM (GIBCO), 100X B27 (GIBCO), 50X N2 (GIBCO), 100X Penicillin–Streptomycin Solution (HyClone), 1 mM sodium pyruvate (GIBCO), 1 mM non-essential amino acids (GIBCO), 1 mM GlutaMAX (GIBCO), 0.1 mM β-mercaptoethanol (GIBCO), 1000 U/ml leukemia inhibitory factor (LIF) (Millipore), as well as the 2i inhibitors, 3 mM CHIR99021 (Sigma) and 1 mM PD0325901 (Sigma). The cells were maintained at 37 °C in a humidified 5% CO_2_ atmosphere, where they grew to form colonies. Culture medium was changed daily, and cells passaged every 3 days to maintain their undifferentiated states.

### Gene knockout methodology

The c-Jun gene in the mESCs was knocked out via the CRISPR-Cas9 system. Cells were transfected with PX330 CRISPR-Cas9 expression plasmids (PX330-2A-PuroR) and PXP plasmids carrying the puromycin resistance gene, both provided by Duanqing Pei’s laboratory (Guangzhou Institute of Biomedicine and Health, Chinese Academy of Sciences). A pair of PX330 CRISPR-Cas9 expression plasmids (PX330-2A-PuroR) within each mESC targeted the regions both up- and downstream from the c-Jun exon, resulting in direct deletion of the c-Jun gene through non-homologous end joining. After puromycin screening to remove non-transfected mESCs, homozygous knockout cell lines were identified by RT-qPCR, and c-Jun protein knockout was verified by Western blot. For co-culturing of c-Jun-ko and wt mESCs, a plasmid, containing the red fluorescent protein (RFP) mCherry and puromycin resistance genes, was transfected into c-Jun-ko mESCs, and puromycin applied to eliminate non-transfected cells.

### *Embryoid body formation and *in vitro* differentiation*

EBs were formed through the hanging drop method [28, 29]. Any adhering mESCs were enzymatically dissociated into single cells using 0.25% Trypsin–EDTA (GIBCO). Cells were then resuspended in differentiation medium, consisting of DMEM supplemented with 20% FBS, 1 mM non-essential amino acids (GIBCO), 1 mM GlutaMAX (GIBCO), 0.1 mM β-mercaptoethanol (GIBCO) and 100X Penicillin–Streptomycin Solution (HyClone). Both wt and c-Jun-ko mESCs were cultured in hanging drops (800 cells per 30 µL differentiation medium), which were then pipetted onto Petri dish lids. These lids were placed back onto 10 cm dishes, containing 10 mL PBS (HyClone), to prevent those hanging drops from drying out. The two groups of mESC (Wt or c-Jun-ko) were used to made EBs (80/group) or examined the corresponding marker on Day 0. Three days later, 20 EBs per group were digested to test the relevant markers and compared. On Day 6, we digested 20 EBs out of the remaining 60 EBs in each group and tested them again for another related marker to compare the differences. After 6 days under hanging drop suspension culture, uniformly-sized EBs were transferred onto glass cover slips, coated with 0.1% gelatin, in a 24-well plate (1 drop per well), and maintained for 3 days (Days 7–9) at 37 °C in a humidified 5% CO_2_ atmosphere, within differentiation medium, for adhesion culturing. After adhesion, the culture medium was changed daily. Each experiment was performed at least three times, in each batch, we will make and differentiate the EBs again.

### Immunofluorescent staining and flow cytometry (FACS) analysis

Both wt and c-Jun-ko cells from adherent EBs on Day 9, growing on the coverslips, were washed 3 times with PBS, fixed with 4% PFA for 30 min, then penetrated and blocked with 0.1% Triton X-100 and 3% BSA for 30 min at room temperature. Cells were incubated with primary antibody (cardiac troponin T [cTnT], cat#565744, 1:300, BD bioscience) for 2 h, washed 3 times in PBS, and incubated with secondary antibody for 1 h. Afterward, cells were incubated in DAPI for 2 min for nuclear staining. The coverslips were then mounted onto the slides for observation under a confocal microscope (LSM-800). For FACS analysis, data acquisition was performed on the Accuri C6 flow cytometer (BD Bioscience) and analyzed using FlowJov X.0.7 software.

### RT-qPCR

Total RNA were prepared with EZ-press RNA Purification Kit (EZBioscience), following the manufacturer’s instructions, and converted to cDNA with ReverTra Ace (Toyobo) reverse transcriptase and oligo-dT (Takara). Gene expression was analyzed by qPCR with Premix Ex Taq (Takara). GAPDH was used as a housekeeping gene, the primers used were presented in Additional file 1: Table S1. Relative expression was calculated using the 2-ΔCt method.

### Western blot

Total protein was extracted from ESCs by lysis buffer. Equal amounts of protein was loaded onto SDS–polyacrylamide gel and transferred to PVDF membranes. After blocking, membranes were incubated with primary antibodies overnight at 4 °C, followed by incubation with HRP-conjugated secondary antibodies. An enhanced ECL western blotting detection reagent was used to detect signals. Antibodies used included c-Jun (Cat#:9165L) and GAPDH (Cat#: KC-5G5) as the loading control.

### Statistical analysis

All values were expressed as mean ± SEM. Statistical analysis was performed with SPSS version 22 software. Independent-samples T tests and nonparametric tests were used for determining statistical significance. *p* < 0.05 was considered statistically significant.

## Results

### Construction and verification of the c-Jun-ko mESC cell line

To study the role of c-Jun in early cardiac development, c-Jun exon was knocked out in mESCs, in accordance to the model shown in Additional file 1: Fig. S1A. c-Jun-ko mESCs were compared to wt mESCs, through PCR and agarose gel electrophoresis, to verify the deletion of the c-Jun exon (Additional file 1: Fig. S1B). Both mESC types were induced to differentiate into ectodermal stem cells (EpiSCs) [21], and no morphological difference found between wt and c-Jun knockout mESCs, confirming successful differentiation (Additional file 1: Fig. S1C). c-Jun mRNA expression in those EpiSCs were also compared, in which no expression was present in the ko group, contrasting with the wt control (Additional file 1: Fig. S1D). This finding was further confirmed at the protein level with Western blot, where c-Jun protein was found to be absent in ko EpiSCs (Additional file 1: Fig. S1E). To determine if c-Jun ko affected the pluripotency of mESCs, RT-qPCR was performed to detect the expression of mouse pluripotency genes (Oct4, Sox2, Nanong, Esrrb, Tfcp2l1) [30, 31] in the 2 mESC groups, and no significant difference for expression of any of those genes were found between the 2 groups (Additional file 1: Fig. S1F). Therefore, we confirmed that c-Jun was successfully knocked out in mESCs, without altering their differentiation and pluripotency capabilities.

To further confirm that no off-targets occurred with the CRISPR-Cas9 system, 2 clones of c-Jun-ko mESCs, #27 and #34, were tested. EBs formed from the 2 clones were found to share the same morphological phenotype (Additional file 1: Fig. S2A) and percentage of differentiated beating cardiomyocytes in Day 6 of differentiation (Additional file 1: Fig. S2B).

### Bulge formation within EBs is associated with cardiomyocyte differentiation

**Fig. 1:**
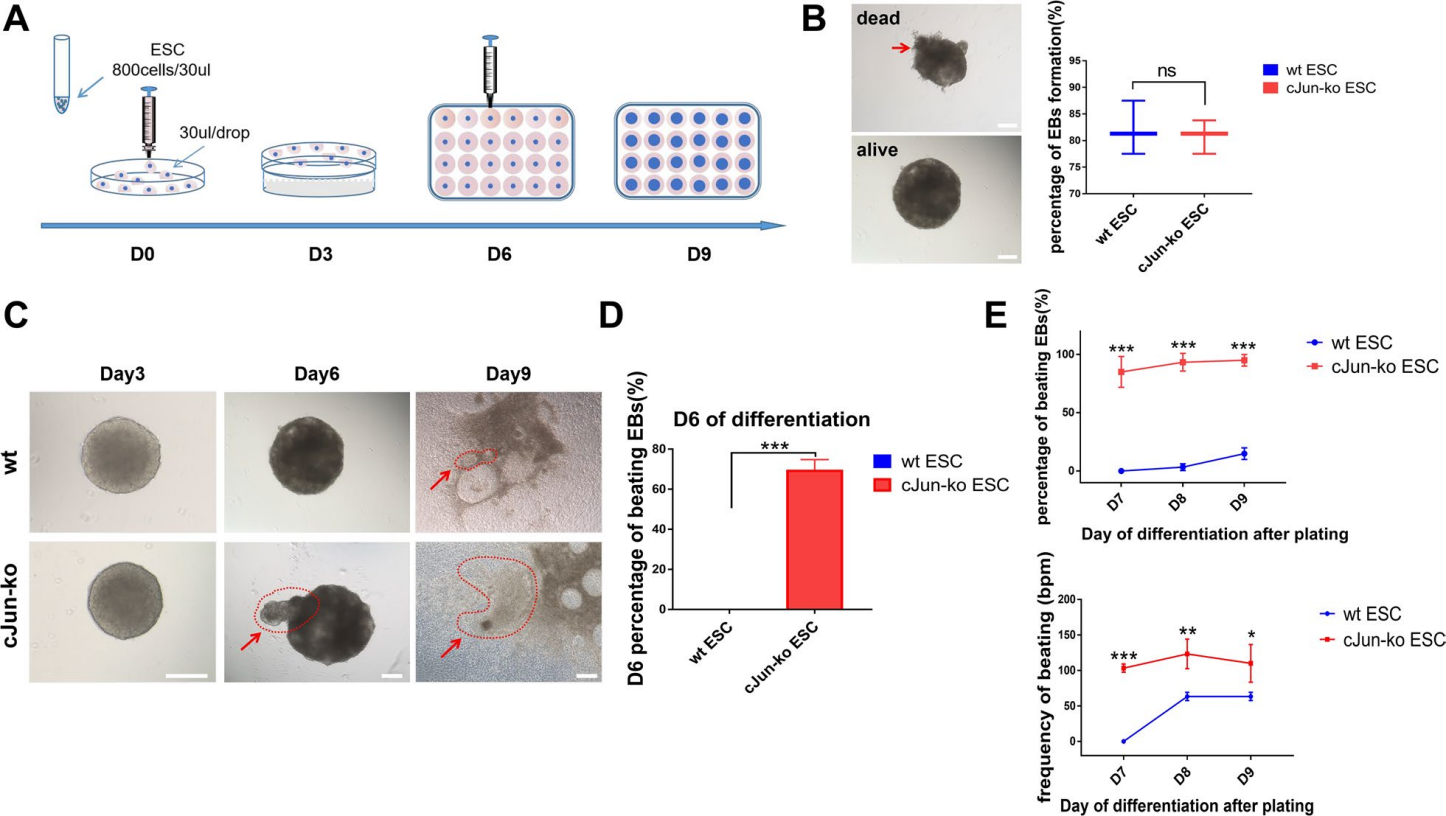
Embryoid body (EB) formation. **A** Schematic diagram of the timeline for EB formation and mESC differentiation. **B** Images of dead and alive EBs, with EB formation percentages for wt and c-Jun-ko ESCs. Scale Bar = 200 µm. **C** Images of EBs for both wt and c-Jun-ko groups in Days 3, 6 and 9 of differentiation. Areas of beating cardiomyocytes are indicated with red arrows. Scale Bar = 200 µm. **D** Percentages of beating EBs for wt and c-Jun-ko ESCs in Day 6 of differentiation. **E** Percentages and frequencies of beating EBs for wt and c-Jun-ko ESCs in Days 7–9 of differentiation. Data represented as mean ± SEM from three independent experiments, *p < 0.05, **p < 0.01, ***p < 0.001, ns denotes no statistical significance, unpaired two tailed student-t-test

To investigate the regulatory role of c-Jun in cardiac development, EBs were formed from both wt and c-Jun-ko mESCs through the hanging drop method, under suspension culture, for 6 days. Afterward, between Days 7–9, EBs were subjected to adherent cell culturing (Fig. 1A). Cell aggregates forming EBs were observed in most hanging drops on Day 6 for both groups, and no significant differences were found for the numbers of successfully formed EBs (Fig. 1B). No significant morphological differences were found between wt and c-Jun-ko EBs on Day 3; however, almost all c-Jun-ko EBs demonstrated a bulging portion in an otherwise smooth sphere on Day 6, which was not present in the wt group (Fig. 1C). The cells comprising that bulge in c-Jun-ko EBs appeared to rhythmically beat when observed under the microscope, which was not present in wt EBs (Fig. 1D, Additional files 2, 3: Videos 1–2). Based on those findings, we postulated that knocking out c-Jun promoted cardiomyocyte differentiation as part of the EB formation process, and that the spontaneous-contracting bulge in c-Jun-ko EBs might be comprised of induced cardiomyocytes. Indeed, a difference in cell differentiation between the 2 groups, after adherence culturing during days 7–9, was observed. Most c-Jun-ko cells continued to adhere to the well and beat like cardiomyocytes under the microscope on Day 7, while beating cardiomyocytes only appeared from Day 8 in the wt. The proportion of beating cardiomyocytes and their beating frequency in c-Jun-ko were significantly higher than for the wt group (Fig. 1E). These findings indicated that c-Jun ko promoted cardiomyocyte differentiation.

### c-Jun knockout promoted EB development into mesoderm and endoderm layers

**Fig. 2:**
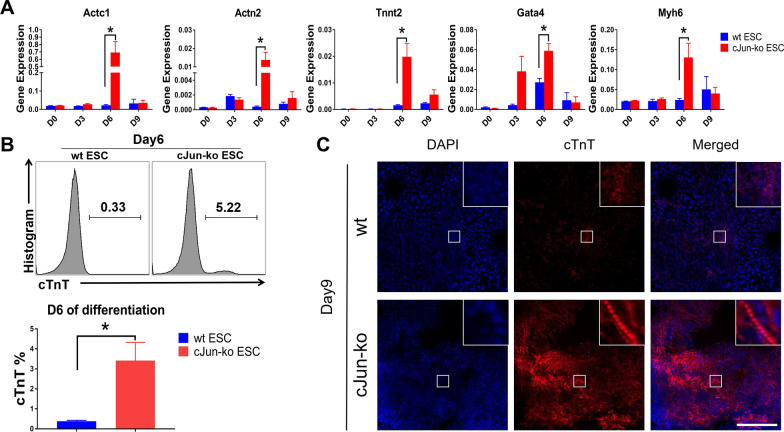
C-Jun knockout promotes mESC differentiation into cardiomyocytes. **A** RT-qPCR results for cardiomyocyte-associated gene expression levels between wt and c-Jun-ko in Days 6 of differentiation. **B** FACS analyses for percentages of cTnT-expressing cells among wt and c-Jun-ko ESCs in Day 6 of differentiation. Note that c-Jun-ko ESCs had a higher percentage (5.22%) of cTnT + cells compared to wt (0.33%). **C** Immunocytochemical staining for cTnT among c-Jun-ko and wt ESCs in Day 9 of differentiation. Insets show high-magnification views, in which obvious sarcomere structures are seen in the c-Jun-ko group. Scale Bar = 200 μm. Data represented as mean ± SEM from three independent experiments, *p < 0.05, unpaired two tailed student-t-test

To further verify whether the c-Jun-ko beating cells observed in EBs on Day 6 were cardiomyocytes, cells from Days 0 (mESCs), 3, 6 (EBs), and 9 (adherent cells) were collected and analyzed by RT-qPCR for expression of specific myocardial markers Actc1, Actn2, Tnnt2, Myh6, and Gata4 [29, 32], with expression levels peaking on Day 6 for the c-Jun-ko group. The expression of those markers on that day was also significantly higher for c-Jun-Ko than for the wt group, indicating that differentiated cardiomyocytes comprised the spontaneously contracting cells within the EB bulge (Fig. 2A). Next, FACS was used to detect the percentage of cTnT + cells, a marker of early cardiomyocyte differentiation, in the Day 6 EBs, and c-Jun-ko EBs had more cTnT + cells present (Fig. 2B). Immunostaining was then performed for cells from both groups on Day 9, revealing significantly higher cTnT in c-Jun-ko compared to the wt group. Additionally, under higher magnification, c-Jun-ko ESCs had obvious sarcomere structures (Fig. 2C), suggesting that knocking out c-Jun promoted cardiomyocyte development in mESCs.

**Fig. 3:**
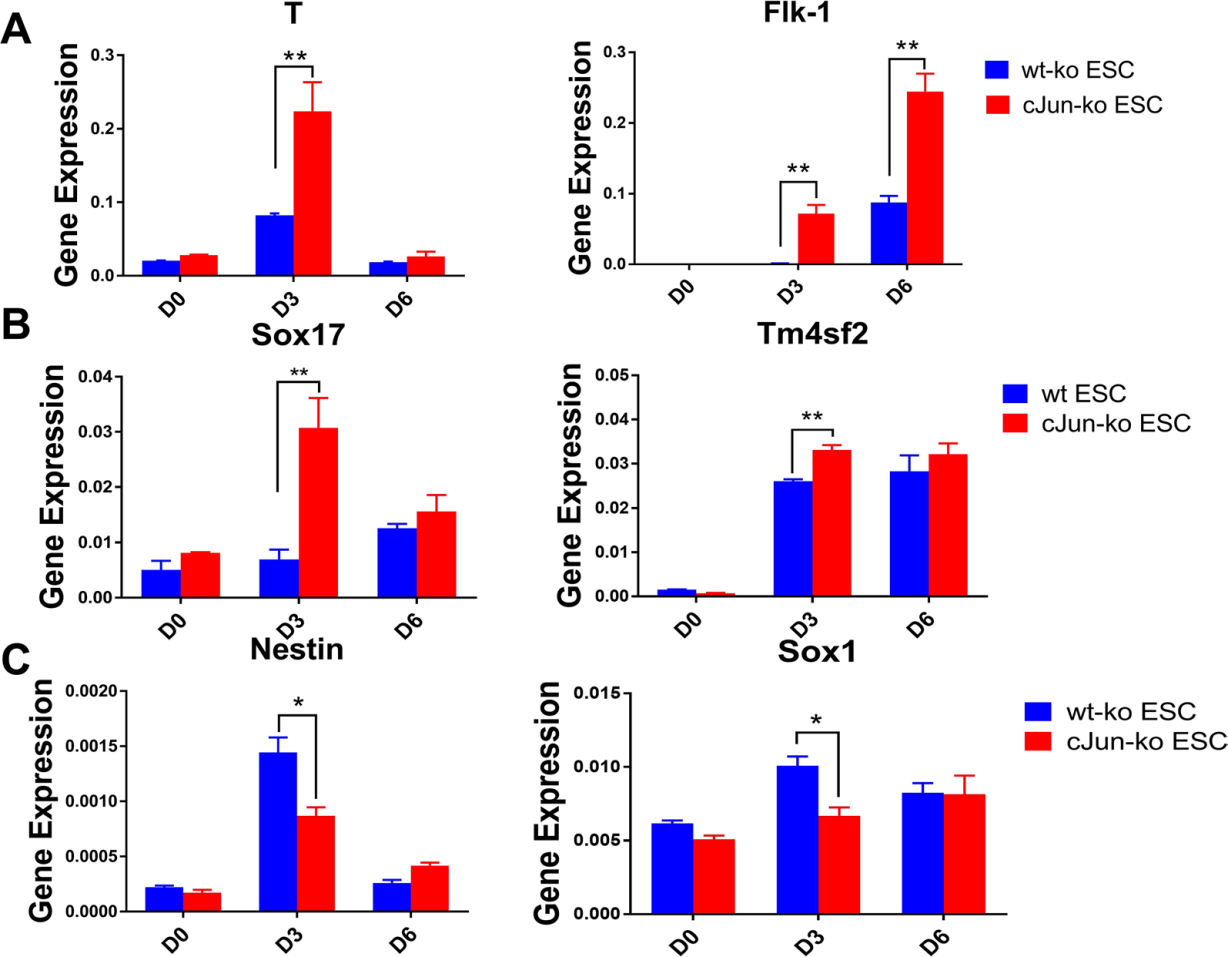
Expression levels for selected mesoderm, endoderm, and ectoderm-associated genes. **A** RT-qPCR results for mesoderm-associated gene expression level between wt and c-Jun-ko ESCs in Days 3 of differentiation. **B** RT-qPCR results for endoderm-associated gene expression level between wt and c-Jun-ko ESCs in Days 0, 3, and 6 of differentiation. **C** RT-qPCR results for ectoderm-associated gene expression level between wt and c-Jun-ko ESCs in Days 0, 3, and 6 of differentiation. Data represented as mean ± SEM from three independent experiments, *p < 0.05, **p < 0.01, unpaired two tailed student-t-test

This promotion of cardiomyocyte differentiation upon knocking out c-Jun was surprising, considering that the normal developmental pathway involves mESCs forming EBs, which then form the 3 germ layers: endoderm, mesoderm, and ectoderm [33]. In light of our finding that Gata4 had significantly higher expression levels on Day 3 in c-Jun-ko ESCs compared to wt, we hypothesized that knocking out c-Jun would promote the development of the mesoderm layer from EBs, followed by cardiomyocyte differentiation. This was owed to Gata4 being found to be upregulated in visceral mesoderm and foregut endoderm during mouse embryonic development [34, 35], as well as the fact that cardiomyocytes are derived from the mesoderm cell layer [13]. To test this hypothesis, EBs from both groups were collected on Days 0, 3, and 6, followed by detection of genes associated with those 3 germ layers. The results showed upregulation of mesodermal markers T, and Flk-1 following c-Jun ko on Day 3 (Fig. 3A), as well as for endoderm markers Sox17 and Tm4sf2 (Fig. 3B). This finding of endoderm-associated gene upregulation upon c-Jun ko is supported by studies showing that the earliest stages of mouse heart formation were dependent on signals from the adjacent endoderm [36]. By contrast, expression of ectoderm markers Nestin and Sox1 were downregulated on Day 3 in c-Jun-ko compared to the wt group (Fig. 3C). Our study therefore suggests that c-Jun-ko promoted early-stage EB differentiation into mesoderm and endoderm, along with inhibiting ectoderm differentiation.

### Co-culture of c-Jun-ko with wt cells proved c-Jun-ko being responsible for further mESC differentiation into cardiomyocytes

**Fig. 4:**
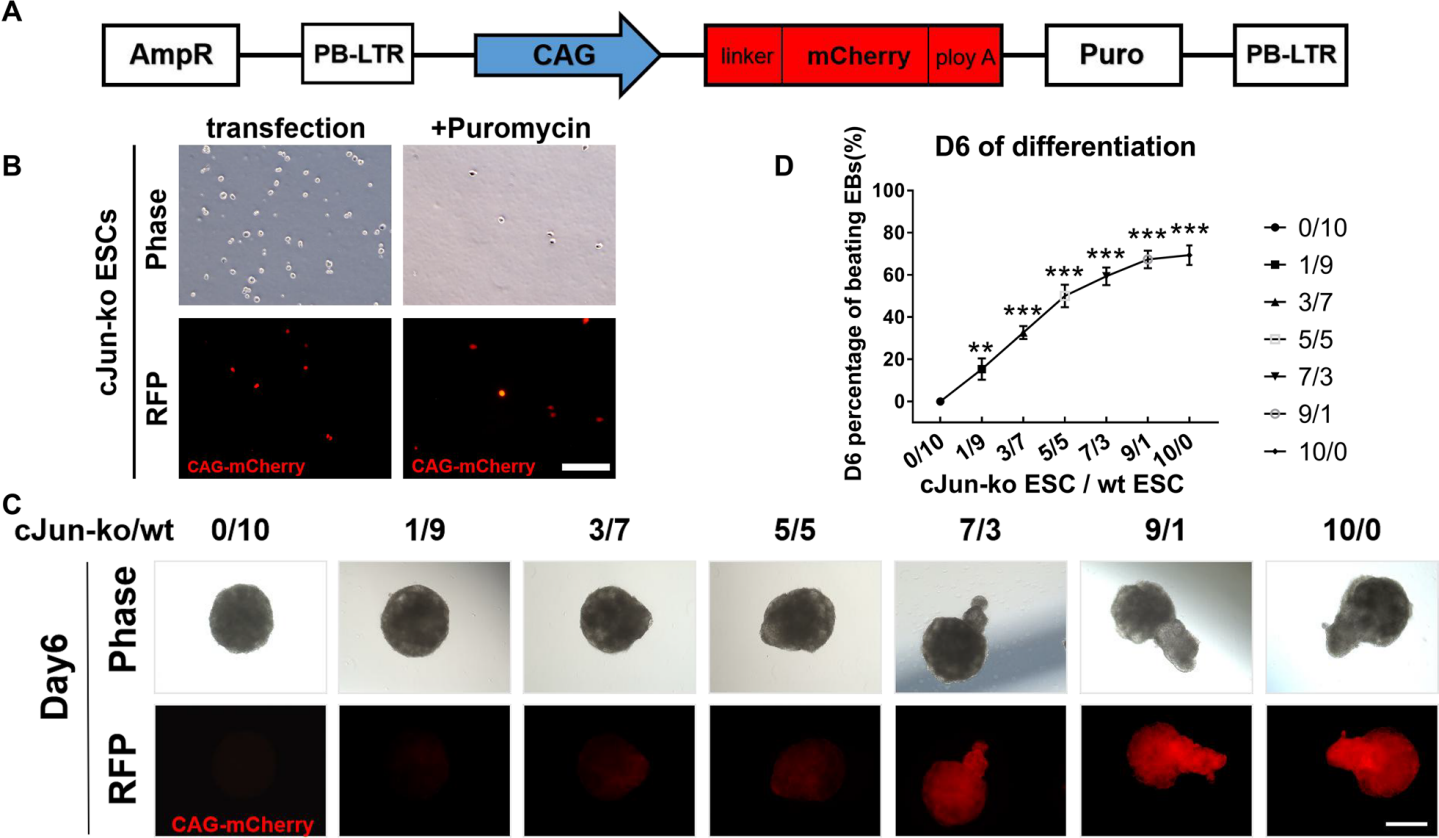
Cardiomyocyte differentiation within co-culture of wt and c-Jun-ko ESCs. **A** Schematic of the plasmid, with mCherry red fluorescent protein and puromycin resistance genes, constructed for the co-culturing experiments. **B** Bright-light and fluorescent images of c-Jun-ko ESCs transfected with the plasmid, before and after cell screening with puromycin. Scale Bar = 200 μm. **C** Images of resulting EBs in Day 6 of differentiation, under both bright-light and fluorescence imaging, with varying proportions of plasmid-containing c-Jun-ko mESCS mixed with wt cells, ranging from pure wt (0/10) to pure c-Jun-ko cells (10/0). Scale Bar = 100 μm. **D** Percentages of beating EBs for varying ratios of c-Jun-ko and wt ESCs, ranging from 0/10 to 10/0, in Day 6 of differentiation. Data represented as mean ± SEM from three independent experiments, **p < 0.01, ***p < 0.001, unpaired two tailed student-t-test

To further validate our finding that knocking out c-Jun promoted cardiomyocyte differentiation, c-Jun-ko and wt mESCs were co-cultured. To distinguish between the 2 cell types, a plasmid containing mCherry and puromycin resistance genes was transfected into c-Jun-ko mESCs (Fig. 4A). Transfected cells, stably expressing mCherry, were then screened via puromycin administration (Fig. 4B), and those mCherry-expressing c-Jun-ko cells were mixed with varying proportions of wt cells to form EBs. Those EBs were observed under the microscope on Day 6, where increasing c-Jun-ko cell proportions corresponded with increasingly prominent convex bulge structures (Fig. 4C). Higher c-Jun-ko cell proportions also correlated with increased percentages of spontaneous beating cells within the co-cultured EBs, to the point where EBs purely comprised of c-Jun-ko mESCs had similar proportions of beating cells as EBs with 9/1 c-Jun-ko/wt cell ratios (Fig. 4D). All of these increases with respect to EB bulges and beating cell percentages corresponded with increased levels of mCherry signaling, in which the strongest signaling was in region with the strongest beating action (Additional files 4, 5, 6: Videos 3–5). These findings further confirmed that c-Jun ko is responsible for mESC differentiation into cardiomyocytes, and that the bulging convex structure formed in EBs is related to cardiomyocyte formation.

## Discussion

In this study, we knocked out the key regulatory gene c-Jun within mESCs to determine its role in cardiomyocyte differentiation. This knocking out did not affect mESC differentiation and pluripotency capabilities, nor did the numbers of EBs differ significantly from wt mESCs. However, it did promote mESC differentiation into cardiomyocytes, without the need to add any additional small molecules into the differentiation medium, as demonstrated by significant upregulation of multiple cardiomyocyte-specific markers on Day 6 in c-Jun-ko cells, along with large numbers of EBs containing beating cardiomyocytes. c-Jun knockout also encouraged the differentiation of EBs into mesoderm and endoderm germ layers, while inhibiting ectoderm differentiation. As vertebrate hearts develop from the mesoderm layer [13], aided by signals from the endoderm layer [36], c-Jun suppression thus play a major role in facilitating cardiomyocyte differentiation. This finding is also supported by the co-culture experiments, where increased proportions of c-Jun-ko cells within mixed-cell EBs were associated with increased percentages of beating cardiomyocytes. Overall, our results showed knocking out c-Jun promoted the formation of mesoderm and endoderm cells in EBs, leading to subsequent differentiation into cardiomyocytes.

As the main component of AP-1, c-Jun has been found to play a significant role in cardiac development and heart disease [18–20]. Previous studies have suggested that inhibiting c-Jun alleviated cardiac hypertrophy through downregulation of cardiac myosin [23]. This alleviation was also observed by Kim-Mitsuyama et al., who demonstrated, through using a dominant negative mutant form of c-Jun to block AP-1 transcription factor binding to angiotensin II, the prevention of cardiac hypertrophy [37]. Therefore, c-Jun blockage inhibits pathologic cardiac hypertrophy. In our study, we found for the first time that c-Jun-ko mESCs were strongly directed into differentiating as beating cardiomyocytes during EB formation, suggesting its involvement in cardiomyocyte development. Our results showed that c-Jun may serve as a negative regulator in early cardiomyocytes, though specific mechanisms still need to be fully defined. Nevertheless, c-Jun could be a new target in developing therapies for heart disease.

Even though various experimental procedures have been developed to promote ESC differentiation into cardiomyocytes, many issues still need to be resolved before applying ESCs in a clinical setting. One issue is that the signaling pathways directing ESC differentiation into cardiomyocytes has not been fully elucidated, as the specific roles and interactions for many key regulatory factors are still unclear. With respect to c-Jun, conflicting results over its role in cardiomyocyte regeneration have been found among different organisms. For instance, some findings show that c-Jun, being highly conserved in somatic cells, resists the reprogramming of those cells into pluripotent stem cells, thereby serving as a maintenance factor for somatic cell fate [21]. These studies are supported by our experiments showing that c-Jun ko significantly increases cardiomyocyte differentiation. We thus speculate that c-Jun may play an important role in maintaining cell fate and terminal differentiation stage of mature cardiomyocytes, preventing their regeneration, in line with adult cardiomyocytes, unlike embryonic ones, being unable to regenerate or self-repair. However, this finding runs counter to another study depicting c-Jun as being required for activating adult killifish cardiomyocyte regeneration [38]. Along these lines, the activation of c-Jun, rather than its inhibition, has been associated with the differentiation of P19 embryonal carcinoma cells into cardiomyocytes, as investigated by Eriksson et al. [39]. There, MAPK signaling leads to differentiation of P19 cells into cardiomyocytes through activating the DNA-binding capabilities of AP-1, which entails increased expression of c-Jun. On the other hand, expression of the dominant negative mutant form of c-Jun inhibits the differentiation process. Both findings were contrary to the results of our study, which may be due to the usage of different cell types from ESCs. Ultimately, whether c-Jun plays either a stimulative or inhibitory role in cardiomyocyte regeneration may be species- and/or cell type-dependent, though it is overall indispensable for the development of multiple organisms. It is likely that its mechanism of action may vary depending on its involved signaling pathways.

## Conclusions

This study reveals the role of c-Jun in early cardiac development, where it serves as a key negative regulator of cardiomyocyte differentiation from mESCs. Its deletion in those cells results in them differentiating along mesoderm and endoderm lineages, with eventual development into cardiomyocytes. Even though the role of c-Jun in vivo is complex, and multiple regulatory factors are involved in cardiomyocyte differentiation, our finding provides new insight into ESC-derived cardiac differentiation. Future studies are needed to elaborate on the role of c-Jun and its related signaling pathways involved in ESC differentiation into cardiomyocytes, to facilitate tissue regeneration for treating cardiac diseases.

### Supplementary Information

**Additional file 1.** Summary of all supplementary information.

**Additional file 2.** Wild type embryoid body (EB) formed from mouse embryonic stem cells (mESC).

**Additional file 3.** C-Jun knockout (ko) EB formed from mESCs, with bulging portion. Cells comprising the bulge portion appeared to rhythmically beat, indicating the presence of cardiomyocyte differentiation.

**Additional file 4.** EB with mCherry+ c-Jun-ko and wt mESCs at 7/3 ratio in Day 6 after differentiation.

**Additional file 5.** EB with mCherry+ c-Jun-ko and wt mESCs at 9/1 ratio in Day 6 after differentiation.

**Additional file 6.** EB with mCherry+ c-Jun-ko mESCs in Day 6 after differentiation.

## Data Availability

All materials and data can be available in the Manuscript and Additional file.

## Abbreviations

mESCs: Mouse embryonic stem cells
ko: Knockout
MI: Myocardial infarction
ESCs: Embryonic stem cells
EBs: Embryoid bodies
wt: Wild-type
RFP: Red fluorescent protein
cTnT: Cardiac troponin T
EpiSCs: Ectodermal stem cells
