# Supplementary material for: The role of c-Jun for beating cardiomycyte formation in prepared embryonic body
Source: Stem Cell Res Ther. 2023 Dec 18;14:371. doi: 10.1186/s13287-023-03544-9 (PMC10729424; doi:10.1186/s13287-023-03544-9)
Supplement: Supplementary file 1 — Additional file 1. Summary of all supplementary information. [file 13287_2023_3544_MOESM1_ESM.docx]

**Supplemental Information**

For

**The role of c-Jun for beating cardiomycyte formation in prepared embryonic body**

**Supplemental Figures**


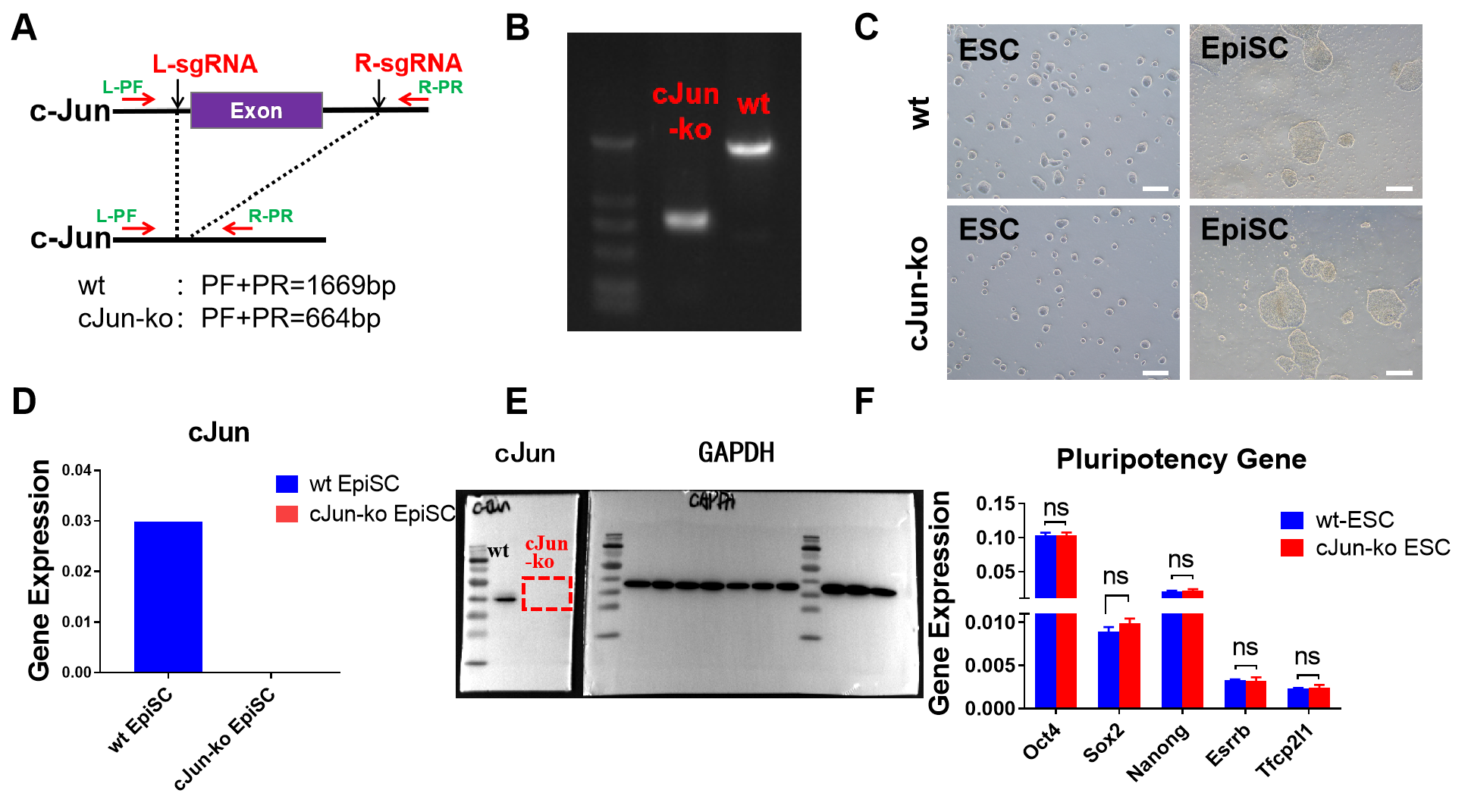


**Supplemental Fig. 1. Construction and verification of c-Jun knockout (ko) mouse embryonic stem cells (mESCs).** A. Schematic diagram for knocking out the c-Jun exon (Purple rectangle). The 2 sgRNAs are highlighted in red, while forward (PF) and reverse primers (PR), used to detect successful c-Jun gene deletion, are in green. B. Gel electrophoresis image demonstrating successful c-Jun gene deletion, with c-Jun-ko (664 bp) and wt (1669bp) alleles. DL2000 DNA Ladder is used. C. No difference in cell morphology was present between both wild-type (wt) and c-Jun-ko ESCs, as well as for ectodermal stem cells (EpiSCs). Scale Bar = 200 µm. D. RT-qPCR results for c-Jun gene expression between wt and c-Jun-ko groups. E. Western blots for c-Jun protein in wt and 2 c-Jun-ko clones of EpiSCs. GAPDH served as a loading control. F. Pluripotency gene expression levels for wt and c-Jun-ko ESCs determined by RT-qPCR. Data represented as mean ± SEM from three independent experiments, ns denotes no statistical significance, unpaired two tailed student-t-test.


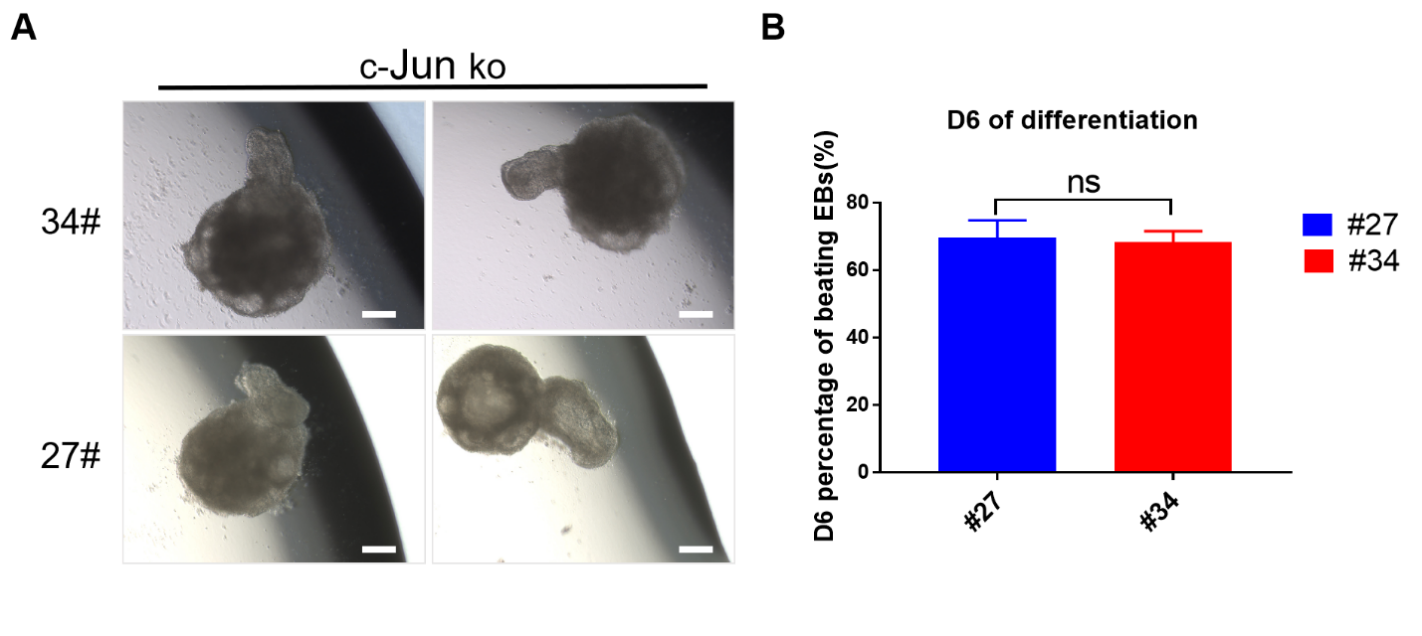


**Supplemental Fig. 2. Two different clones of c-Jun knockout (ko) cells, #27 and #34, formed embryoid bodies (EBs) in Day 6 of differentiation.** A. Both clones had the same morphological phenotype, with spontaneously beating cardiomyocytes within the convex bulges. Scale Bar = 200 µm. B. No statistically significant difference was found between the percentages of beating cells within the EBs of both clones on Day 6. Data represented as mean ± SEM from three independent experiments, ns denotes no statistical significance, unpaired two tailed student-t-test.


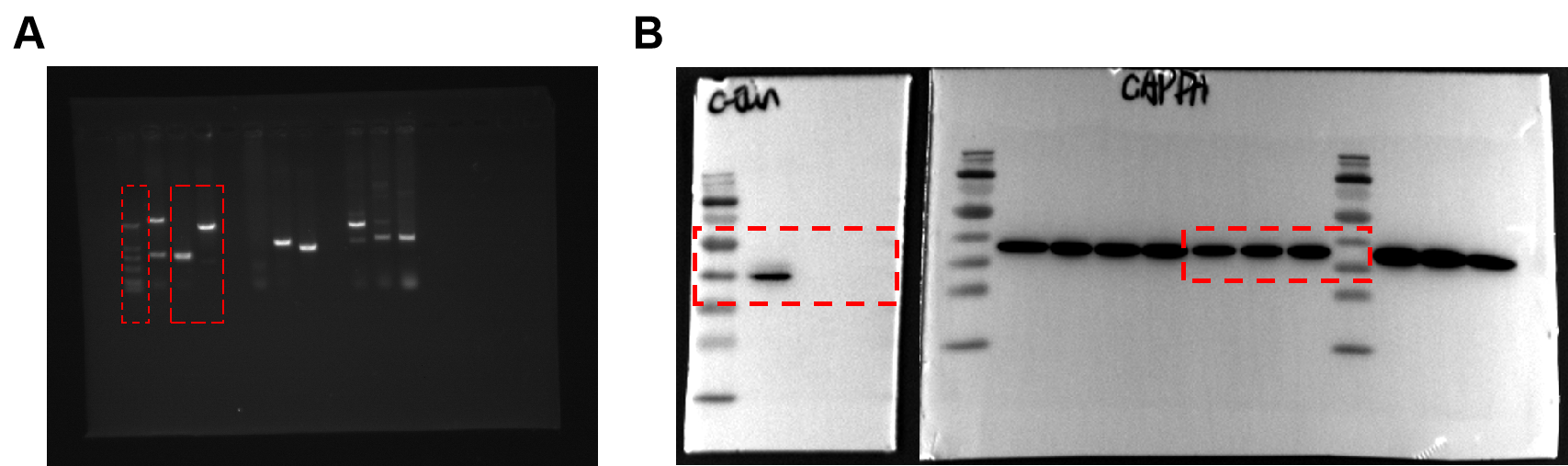


**Supplemental Fig. 3. Full-length blots/gels of our experiments.** A. Full-length gel electrophoresis image demonstrating successful c-Jun gene deletion. Cropped along the red dashed line. B. Full-length blots for c-Jun protein in wt and 2 c-Jun-ko clones of EpiSCs. GAPDH served as a loading control. Cropped along the red dashed line.

**Supplemental Videos**

**Supplemental Video 1.** Wild type embryoid body (EB) formed from mouse embryonic stem cells (mESC).

**Supplemental Video 2.** C-Jun knockout (ko) EB formed from mESCs, with bulging portion. Cells comprising the bulge portion appeared to rhythmically beat, indicating the presence of cardiomyocyte differentiation.

**Supplemental Video 3.** EB with mCherry^+^ c-Jun-ko and wt mESCs at 7/3 ratio in Day 6 after differentiation.

**Supplemental Video 4.** EB with mCherry^+^ c-Jun-ko and wt mESCs at 9/1 ratio in Day 6 after differentiation.

**Supplemental Video 5.** EB with mCherry^+^ c-Jun-ko mESCs in Day 6 after differentiation.

**Supplemental Tables**

**Supplemental Table 1.** Primer sequences used for RT-qPCR.

| Primer | Forward | Reverse |
| --- | --- | --- |
| GAPDH  c-Jun  Oct4  Sox2  Nanong  Esrrb  Tfcp2l1  Actc1  Actn2  Tnnt2  Gata4  Myh6  T  Flk-1  Fgf8  Sox17  Tm4sf2  Nestin  Sox1 | AACTTTGGCATTGTGGAAGGGCTCA  AAGATGGAAACGACCTTCTA  TAGGTGAGCCGTCTTTCCAC  AGGGCTGGGAGAAAGAAGAG  CTCAAGTCCTGAGGCTGAC  TTTCTGGAACCCATGGAGAG  GCTGGAGAATCGGAAGCTAGG  TCTGGCACCATACATTCTAC  GATGGCAATGTGAAGATGAC  GTGAGGAGGAGGAGAACA  CAGCAGCAGTGAAGAGAT  GAACACCAGCCTCATCAA  TCTCCAACCTATGCGGACAAT  TGGAGGAAGAGGAAGTGT  GCTCATTGTGGAGACCGATAC  CAGTATCTGCCCTTTGTGTA  CCAGTTGCTGCATGAACGAA  TCCCTTAGTCTGGAAGTGGCTA  GTGACATCTGCCCCCATC | TTGGCAGCACCAGTGGATGCAGGGA  GGTTACTGTAGCCGTAGG  GCTTAGCCAGGTTCGAGGAT  CCGCGATTGTTGTGATTAGT  TGAAACCTGTCCTTGAGTGC  AGCCAGCACCTCCTTCTACA  AAAACGACACGGATGATGCTC  AGGCACATTGAAGGTCTC  GGTGTGGAAGTTCTGGATAT  CTCTTCCGCTCTGTCTTC  GTCTGAGTGACAGGAGATG  TGCCTCCTCTACTTCTGT  TAGGTGGGCTGGCGTTATGA  GTCTGTCTGGCTGTCATC  TTGCTCTTGGCAATTA GCTTC  GCAATAGTAGACCGCTGAG  CACCAGATCATAACAG CCCTTCT  GGTGTCTGCAAGCGAGAGTT  GAGGCCAGTCTGGTGTCAG |
